# Putative Transcriptional Regulation of HaWRKY33-AOA251SVV7 Complex-Mediated Sunflower Head Rot by Transcriptomics and Proteomics

**DOI:** 10.3390/plants14193018

**Published:** 2025-09-29

**Authors:** Qian Zhang, Xin Wang, Guoyu Fu, Meishan Zhang, Xueyu Leng, Zicheng Kong, Jing Wang, Yanjie Zhang, Xiaoxin Hu, Huan Yu, Zhongchen Zhang

**Affiliations:** 1 College of Agriculture, Northeast Agricultural University, Harbin 150030, China; 17354616769@163.com (Q.Z.); 15765222963@163.com (X.W.); 18324580654@163.com (G.F.); Zzhangmeishan@163.com (M.Z.); 17561746702@163.com (X.L.); kzc020301@163.com (Z.K.); 18747626039@163.com (X.H.); 2 Gannan County Sunflower Technical Service Center, Qiqihar 162100, China; wangjing1983320@163.com (J.W.); laoda.2.1.5@163.com (Y.Z.)

**Keywords:** sunflower head rot, *HaWRKY33*, simple sequence repeat, transcriptome analysis, AlphaFold-guided redesign

## Abstract

*HaWRKY33* is induced by salicylic acid and participates in the disease resistance signaling pathway of sunflower rust disease; however, the transcriptional regulatory mechanism of this protein against *Sclerotinia sclerotiorum* in sunflowers remains unclear. Given this, we conducted a survey of 426 sunflower accessions at the natural disease nursery in Gannan County and identified a single dominant physiological race, MCG1, using simple sequence repeat methods. Additionally, we performed indoor inoculation tests using this dominant race and obtained disease-resistant varieties, W227 and BC2202-03, as well as susceptible varieties, N241 and Z155. Further, we inoculated the above resistant and susceptible combination materials with MCG1 and conducted transcriptomic analysis and RT-qPCR validation. Through KEGG analysis, we found that *HaWRKY33* is involved in the plant–pathogen interaction pathway, suggesting that *HaWRKY33* may regulate sunflower defense responses against *Sclerotinia sclerotiorum* through the plant–pathogen interaction pathway. Finally, yeast two-hybrid screening and AI prediction using AlphaFold 3 revealed strong interactions between ARG-189 and GLU-344 amino acids in the HaWRKY33-AOA251SVV7 proteins, indicating that the HaWRKY33-AOA251SVV7 pattern regulates the sunflower defense response against *Sclerotinia sclerotiorum* in a transcriptional complex form. In summary, these results provide new insights into the disease resistance mechanisms of sunflowers against *Sclerotinia sclerotiorum* and promote the development of molecular breeding for sunflower resistance to *Sclerotinia sclerotiorum*.

## 1. Introduction

Sunflower (*Helianthus annuus* L.) is one of the most important oil crops in the world [1]. It possesses strong stress resistance, drought resistance, and salt tolerance [2,3]. According to the Food and Agriculture Organization of the United Nations, the global sunflower seed production in 2022 reached approximately 50.2 million tons, with a harvest area of about 27.8 million hectares. China is the fourth-largest sunflower seed producer, with an annual output of 2.375 million tons. Sunflowers are currently cultivated in 19 provinces in China, with their primary growth areas located in northern China. Heilongjiang Province, one of the main producing areas of sunflowers, in Gannan County in Qiqihar, was named the Hometown of Chinese Sunflower in 2000 [4].

Discomycete fungi *S. sclerotiorum* (Lib.) de Bary is a highly destructive pathogen that causes severe diseases in sunflowers. *S. sclerotiorum* has a wide host range of more than 400 plants, including important crops like sunflowers, oilseed rape, soybean, and lentils [5]. Moreover, some weeds are considered fungal breeders. *S. sclerotiorum* generally survives in soil for 4–5 years but can survive for up to 8 years [6,7]. Heilongjiang Province is an important production base for sunflowers; however, by the end of the 20th century, its sunflower-producing areas decreased by about 30–60% due to *S. sclerotiorum*, reaching more than 90% in some areas, causing great losses in sunflower yields. In recent years, the introduction of a large number of domestic and foreign species in some areas in Heilongjiang Province has resulted in an increase in the incidence of the disease year by year, seriously restricting the yield of sunflowers [8]. Therefore, selecting and breeding disease-resistant varieties is key to solving the problem of sunflower head rot.

Biogenetic diversity, also known as genetic diversity, refers to the difference that exists at the molecular level between individuals of each species or population in nature [9]. Because of the wide host range of *S. sclerotiorum*, the existence of a large number of polymorphisms between strains from different hosts, and strong resistance, it is difficult to identify strains using conventional identification methods [10,11]. With the rapid development of molecular biotechnology in recent years, genetic diversity research in molecular biology has become the most common and effective analysis method. The study of genetic diversity in *S. sclerotiorum* mainly involves the mycelial affinity group (MCG), which is used to determine genetic relationships among fungal strains. It works by observing whether hyphae from different strains fuse upon contact (or form an antagonistic zone). Based on this observation, the affinity groups to which the strains belong can be distinguished. This aids in analyzing fungal population structure, transmission pathways, or pathogenicity differences. A microsatellite marker, also known as a simple sequence repeat (SSR), is a short sequence composed of 2–6 nucleotides (e.g., “ATATAT” and “GCGCGC”) that repeats multiple times within a genome. Due to significant variation in the number of repeats across individuals or strains, these sequences are commonly used as molecular markers for studies such as species genetic diversity analysis, genotype identification, or gene mapping. When different strains belonging to the same mycelial affinity group are mixed with each other, they can grow to form a uniform colony. When different strains belonging to two different mycelial affinity groups are mixed, their mycelia interact with each other in an antagonistic manner, leading to a large number of deaths, which result in clear demarcation lines; this phenomenon mainly manifests as reduced growth, cell death, or thinning of the mycelium [12]. Silva et al. (2021) characterized the mycelial affinities of 238 nucleated discomycetes from different hosts in Brazil and obtained a total of 22 MCGs, of which 8 new MCGs were found [13]. Liu et al. divided 115 strains of sunflower nucleocapsid collected in Northeast China into 35 MCGs, of which 9 MCGs were composed of single strains, indicating that the affinity variation among strains from different regions was large and that there was genetic diversity and population variability [14]. Up to now, many studies have characterized the affinities of *S. sclerotiorum* and have found significant disaffinities between *S. sclerotiorum*, and these results have demonstrated that genetic diversity exists within *S. sclerotiorum* populations and also demonstrated that the determination of *S. sclerotiorum* affinities can be used as a method of differentiating between strains of strain specificity [15].

Transcription factors, also known as trans-acting factors, are important regulators that bind specifically to the cis-elements upstream of target gene promoters and play a key regulatory role in activating or repressing transcription of their target genes [16]. In the current study, a variety of transcription factors have been identified in higher plants, which are generally composed of four components: DNA-binding structural domains, oligomeric structural domains, transcriptional regulatory structural domains, and nuclear localization signaling regions [17,18]. Six of them are involved in plant immune responses to external stimuli and biotic stresses: AP2/ERF, bHLH, MYB, NAC, WRKY, and bZIP. Transcription factors play important roles in regulating plant resistance responses to disease when plants defend themselves against pathogenic bacteria. It was found that transgenic soybean plants overexpressing *GmbZIP15* increased resistance to Botrytis cinerea and soybean blight. In addition, *GmbZIP15* regulates soybean response to pathogenic bacteria by modulating the antioxidant defense system and phytohormone signaling [19]. In rice, *OsWRKY10* can directly or indirectly bind to the W-box motif of the *OsPR1a* promoter by interacting with *OsWRKY47* to regulate the expression of the *OsPR1a* gene, thereby enhancing the resistance of rice to white leaf blight [20]. Li concluded that the expression of the *HaWRKY33* gene in sunflower was strongly induced by rust infestation. The expression of the *HaWRKY33* gene was significantly up-regulated in the early stage under the condition of inoculation with rust after exogenous salicylic acid treatment. It was hypothesized that salicylic acid treatment could induce the expression of *HaWRKY33* in the disease resistance signaling pathway [21]. While prior research has provided valuable insights into the role of *HaWRKY33* in sunflower rust resistance, significant gaps persist regarding its involvement in defense against *S. sclerotiorum*. To address these gaps, the present study aims to achieve the following: (1) investigate the transcriptional regulatory network of *HaWRKY33* in response to *S. sclerotiorum* infection, with a focus on identifying upstream regulators and downstream target genes; and (2) perform a predictive analysis of key amino acid residues critical for antagonistic function in *HaWRKY33*. By addressing these questions, this study aims to provide a more comprehensive understanding of the molecular basis of HaWRKY33-mediated resistance to *S. sclerotiorum* in sunflowers, thereby laying the groundwork for future breeding strategies that enhance disease resistance.

In sunflowers, the *HaWRKY33* gene plays a key role in the regulation of disease resistance. Therefore, in this study, we constructed a yeast two-hybrid cDNA library, screened the proteins that might interact with the HaWRKY33 protein, and based on that, we used AlphaFold 3 3.0.1 (accessed on 15 March 2025 via https://alphafoldserver.com/) to predict protein interactions, calculated the pTM + ipTM values, and screened the proteins whose values were greater than 0.75. We further deepened and verified the screening results and visualized them using PyMOL (accessed on 21 March 2025 via https://pymol.org/) to identify key amino acid sites involved in protein interactions. Subsequently, we performed bioinformatic mutation simulation to induce extreme structural changes in the protein, thereby revealing critical aspects of its functional mechanisms and providing a foundation for future research on enhanced disease resistance. It is anticipated that the results were further deepened and verified through a series of steps. The objective of this investigation was to elucidate the structural basis of protein–protein interactions and identify critical functional residues through a combined computational–experimental approach. Specifically, by visualizing the data with PyMOL, we can identify the amino acid sites where the proteins interact with each other and then conduct bioinformatic mutation simulation to reveal key protein functions by maximizing the structural differences among proteins after mutagenesis. These measures are hypothesized to provide an important foundation for the future exploration of the gene *HaWRKY33* resistance to *S. sclerotiorum* in sunflower. However, these predictive claims are subject to additional experimental validation and logical scrutiny to ensure their accuracy and reliability.

## 2. Results

### 2.1. Identification of Sunflower Resistance Resources Against S. sclerotiorum

#### 2.1.1. Field Identification of Sunflower Head Rot Disease

**Figure 1:**
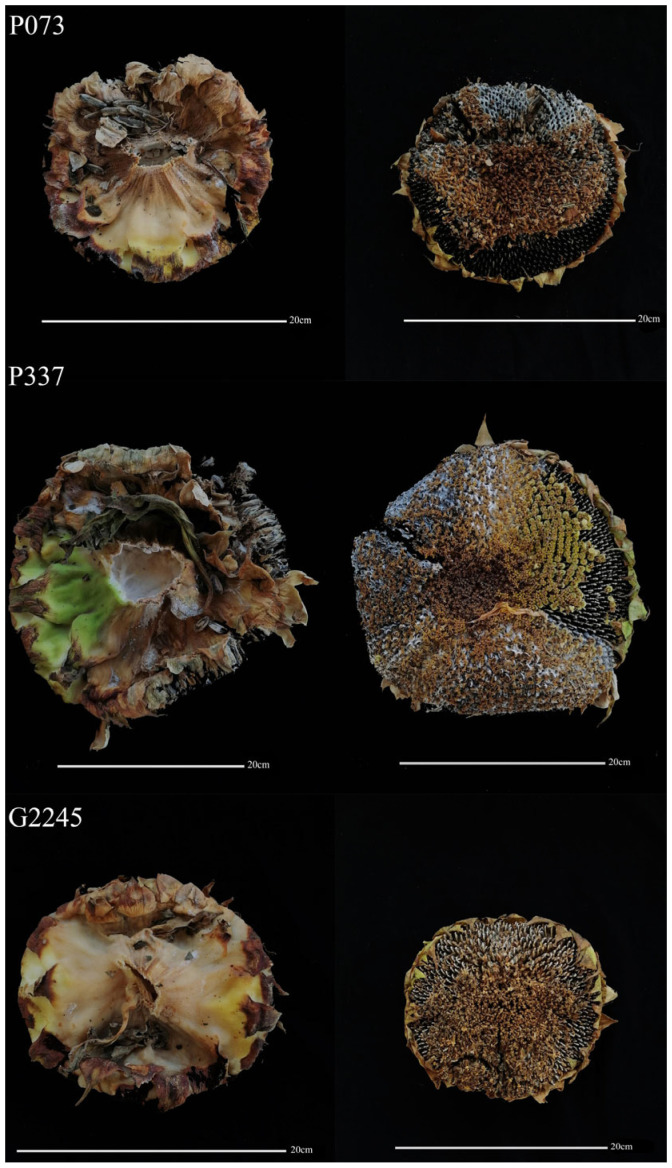
Field identification of sunflower against S. sclerotiorum. Resistant (G2245), moderately resistant (P073), and susceptible (P337).

We collected 426 sunflower lines and planted them in the experimental field of Gannan County in the spring of 2022. In September 2022, we conducted a statistical analysis of the degree of disease in the field. The statistical results showed that among the 426 sunflower materials planted in the field, 271 were immune, 54 were moderately resistant, 80 were resistant, 15 were susceptible, and 6 were moderately susceptible (Figure 1).

#### 2.1.2. Identification of the Affinity of *S. sclerotiorum* Mycelium Toward Sunflowers

**Figure 2:**
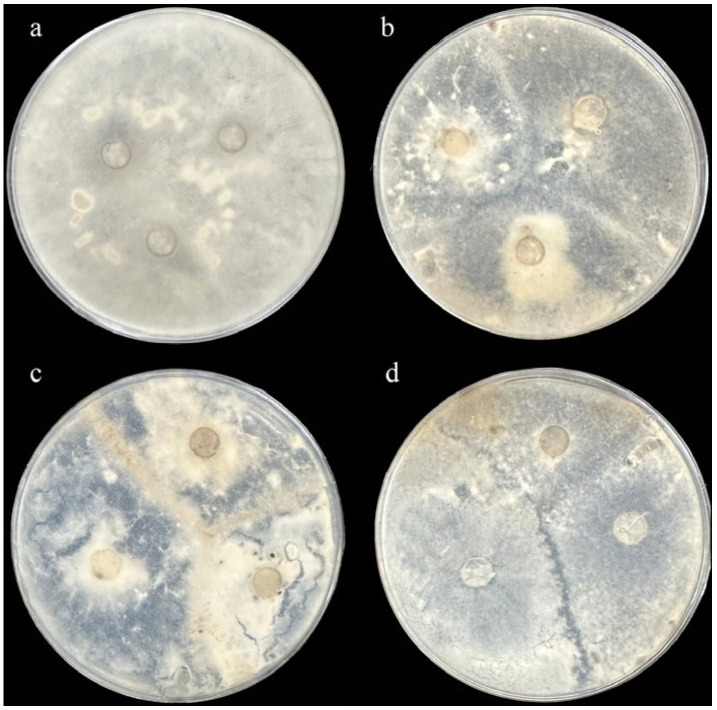
Mycelial interactions between different isolates of sunflower head rot were tested on PDA medium. (**a**) Mycelial compatibility of the same isolate as the positive control. (**b**) Fusion of the three colonies indicates compatibility of the three isolates. Incompatible isolates producing an obvious barrage zone (**c**) or a region of sparse mycelia on PDA medium (**d**).

A total of 66 *S. sclerotiorum* isolates of sunflower head rot were obtained from Gannan County. We performed MCGs analysis based on mycelial compatibility groups (Figure 2). The pairing results are either compatible or incompatible. In the contact zone, the mixing of paired mycelium was accompanied by anastomosis (i.e., the fusion of two colonies), indicating that the two isolates were compatible (Figure 2a,b), while the incompatible isolates produced obvious closed zones or sparse mycelium (Figure 2c,d). We identified 6 different MCGs from 66 isolates of sunflower head rot (Table 1). The largest MCG1 contained 21 isolates, and the other MCGs had 7–11 isolates.

#### 2.1.3. SSR Analysis Reveals the Genetic Diversity of *S. sclerotiorum* Populations in Sunflower

We tested 25 SSR markers (Figure S1) and found that five groups did not produce amplification products (AF377899, AF377903, AF3779905, AF377906, and AF377917). The remaining 20 pairs of primers produced a single band (sized between 100 and 500 bp) on agarose gel electrophoresis, which we used to identify the genetic structure of sunflower head rot. Denaturing polyacrylamide gel electrophoresis analysis showed distinguishable bands and allele polymorphisms (Figure S2). The statistical analysis indicated that these 20 pairs of primers could theoretically detect 84 microsatellite loci, and the primer pair AF377921 had up to 8 alleles, with an average of 4.2 alleles per locus. Analysis of 66 isolates using 20 molecular markers yielded 74 polymorphic amplified fragments, resulting in an average polymorphic rate of 88.1% (Table S1). These findings suggest that the selected primers are effective in detecting highly polymorphic microsatellite loci.

**Figure 3:**
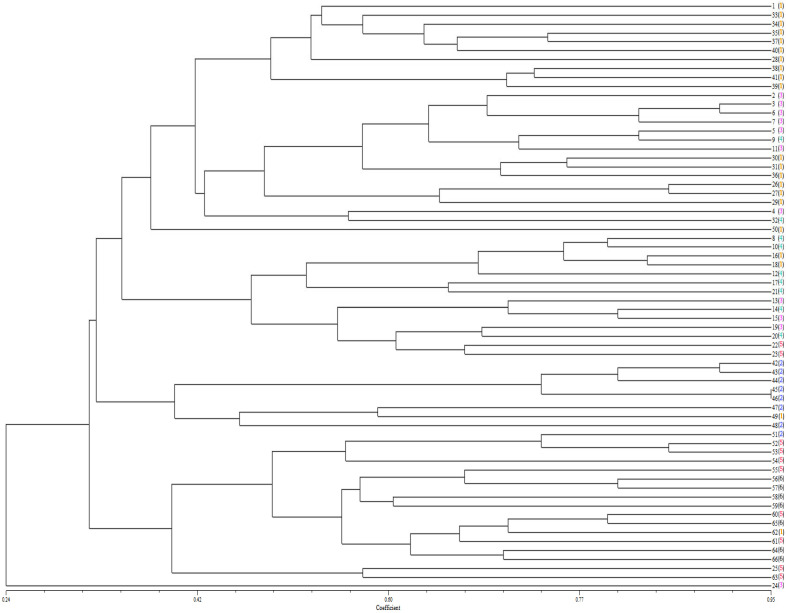
Cluster analysis of 66 S. sclerotiorum isolates based on SSRs data. The lateral black numbers in front of the bracket symbol are 1–66 isolates of S. sclerotiorum, and the numbers of different colors in the bracket represent different MCGs. In the figure, the brown, blue, pink, green, red, and black numerals represent strains assigned to MCG1, MCG2, MCG3, MCG4, MCG5, and MCG6, respectively.

We performed cluster analysis on 66 sunflower sclerotium rot strains using NTSYS software. The clustering results revealed rich genetic diversity among strains, with a certain association between MCGs and SSR (Figure 3). For example, strains 42, 43, 44, 45, and 46 belonged to the same branch in the clustering results and were also grouped in MCG2 in the affinity group pairing. Strains 1, 28, 33, 34, 35, 37, 38, 39, 40, and 41 also belonged to the same branch in the clustering results and were grouped in MCG1 in the affinity group pairing. This finding aligns with other reports [22,23]. However, there is also a phenomenon where strains of MCG2, MCG3, MCG4, and MCG5 are widely scattered in the SSR clustering tree. We propose that the reasons for this partial consistency yet overall dispersion may be as follows: First, SSR molecular markers detect randomly distributed loci across the entire genome, while the classification of mycelial compatibility groups (MCGs) is based on the ability of hyphal fusion under specific physiological conditions. These two methods reflect genetic relationships from different perspectives—SSRs represent the similarity of the overall genetic background, whereas MCGs focus more on genetic differences related to hyphal fusion and vegetative compatibility. Such differences in detection dimensions make it difficult for them to achieve complete correspondence. Second, the population of 66 sunflower sclerotium rot strains used in this study has a complex genetic structure. Factors such as genetic recombination and variation may lead to changes in local gene loci among strains with close genetic relationships, resulting in different performances in hyphal compatibility detection and thereby disrupting the neat correspondence between SSR clustering and MCG grouping.

In summary, by integrating the results of SSR clustering analysis and MCG identification, we found that MCG1 contains 21 *S. sclerotiorum* isolates, which also cluster within the same major branch in the analysis. Therefore, we hypothesize that the isolates within MCG1 represent the locally dominant race, and this dominant race will be used in subsequent in vitro inoculation identification tests.

#### 2.1.4. Indoor Identification of Sunflower Head Rot Disease

**Figure 4:**
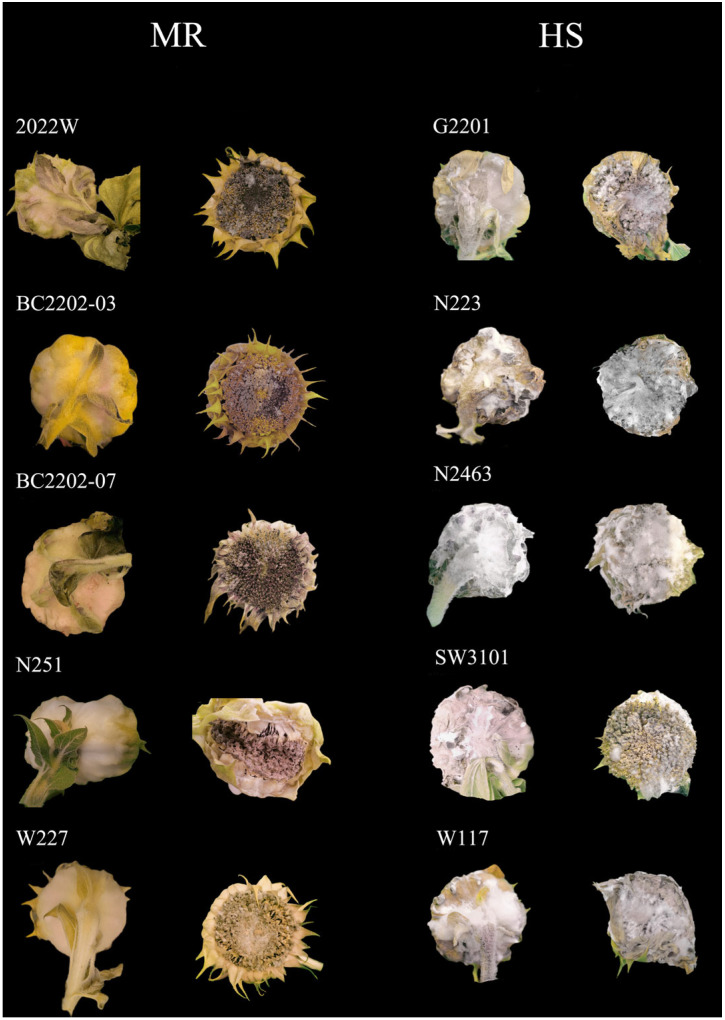
Moderately resistant and highly susceptible sunflower lines. MR lines: 2022W, BC2202-03, BC2202-07, N251, and W227. HS lines: G2201, N223, N2463, SW3102, and W117.

Due to the low disease incidence in the field, we selected immune and resistant lines with strong stems and high-yielding traits for indoor inoculation experiments. A total of 148 lines were selected for planting in an artificial climate chamber. We inoculated the dominant race (MCG1), speculated by MCGs and SSRs analysis, on the head of the sunflower. The results showed that 143 of 148 sunflowers showed high susceptibility, and 5 showed moderate resistance. Most of the lines showing immunity, moderate resistance, and resistance in the field were transformed into high susceptibility (Table S2). Finally, we identified 2022W, BC2202-03, BC2202-07, N251, and W227 as medium-resistant lines based on the results of field investigation and indoor inoculation identification (Figure 4).

### 2.2. Transcriptome Analysis of Sunflower Disease-Resistant and Susceptible Varieties in Response to S. sclerotiorum Infection

#### 2.2.1. GO and KEGG Enrichment Analysis of Differentially Expressed Genes

Through comparative transcriptome analysis, studies have identified differentially expressed genes (DEGs) in resistant and susceptible sunflower genotypes after inoculation with Sclerotinia sclerotiorum. The methodologies and findings offer valuable reference for investigating the molecular mechanisms of defense responses to *S. sclerotiorum* and for breeding sunflower varieties resistant to this pathogen [24]. Transcriptome sequencing was performed on two resistant sunflower varieties (W227 and BC2202-03) and two susceptible varieties (N241 and Z155). Protein-coding gene expression levels were quantified across samples, and differential expression analysis was conducted using DESeq2. DEGs were defined as genes meeting thresholds of q-value < 0.05 and |fold change| > 2. A total of 9443 DEGs were identified between resistant and susceptible varieties post-inoculation. Among these, 6170 genes were up-regulated and 3273 genes were down-regulated in resistant cultivars compared to susceptible controls.

Gene Ontology (GO) analysis assigned functional annotations to 6763 DEGs, yielding 3114 unique GO terms. Enriched terms were filtered using a threshold of PopHits ≥ 5 and ranked by significance (-log_10_ *p*-value). The top 30 enriched GO terms spanned three categories: biological processes, cellular components, and molecular functions (Figure S3a,b). Subsets highlighting the top 10 terms by -log_10_ *p*-value are shown in Figure S3c,d. Comparative analysis revealed distinct functional profiles between up-regulated and down-regulated genes in resistant varieties following inoculation.

Under conditional selection criteria, DEGs were enriched in transcriptional regulation, abscisic acid response, protein ubiquitination, defense response, and salt stress pathways (Figure S4). Up-regulated DEGs showed specific enrichment in cell wall organization (140 genes), cell division (107 genes), carbohydrate metabolism, drought response, and bacterial defense mechanisms. Conversely, down-regulated DEGs were associated with antioxidant responses, cadmium ion response, ethylene-activated signaling, cold stress adaptation, and plant-type hypersensitivity reactions.

Cellular component analysis revealed comparable enrichment patterns for both regulation directions, with shared terms including nucleus, membrane systems (plasma membrane and integral membrane components), cytoplasm, chloroplasts, cytosol, plasmodesmata, and extracellular regions. Notably, up-regulated genes exhibited Golgi apparatus enrichment, while down-regulated genes showed mitochondrial preference.

At the molecular function level, common enriched terms included metal ion binding (particularly iron and zinc), ATP binding, DNA-binding transcription factor activity, sequence-specific DNA binding, protein serine/threonine kinase activity, and heme binding. Regulation-specific differences emerged with up-regulated genes enriched for microtubule binding and protein dimerization activity, versus down-regulated genes showing zinc ion binding and oxidoreductase activity.

**Figure 5:**
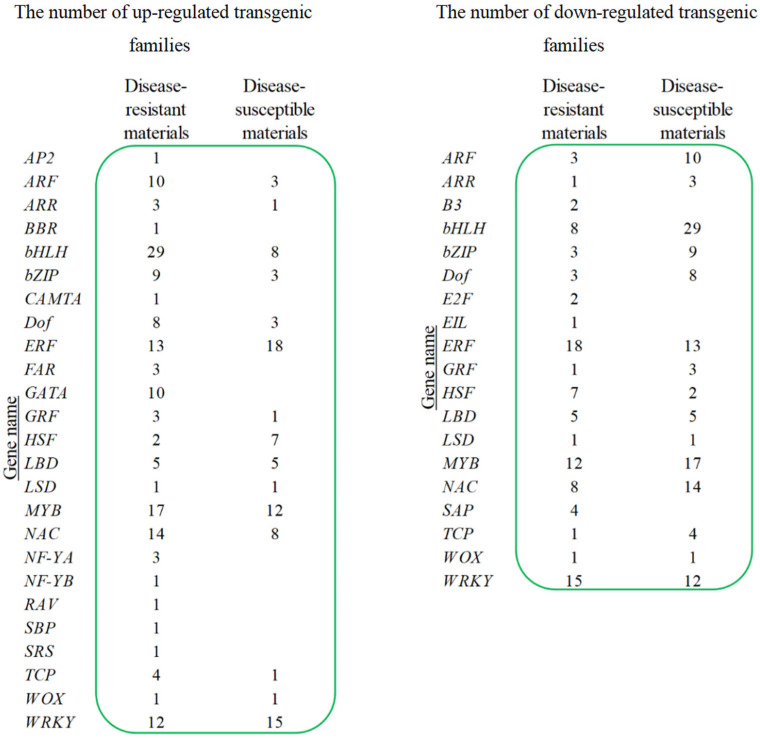
The number of up-regulated and down-regulated transgenic families.

We identified 29 gene families encoding transcription factors, which comprised a total of 250 genes. Comparative analysis revealed differential expression patterns between disease-resistant and susceptible materials (Figure 5). In resistant materials, 154 genes were up-regulated and 96 down-regulated, while in susceptible materials, 87 up-regulated and 131 down-regulated genes were detected.

**Figure 6:**
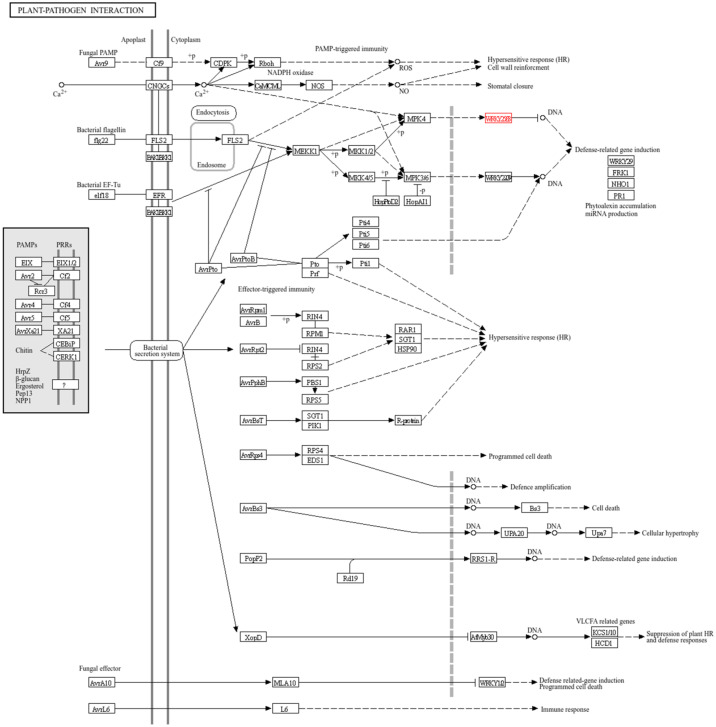
Plant–pathogen interaction pathways for HaWRKY33. In this figure, the graphical conventions are defined as follows: a solid arrow (—>) denotes a catalytic reaction or direct activation; a dashed arrow (-->) indicates an indirect effect or regulatory relationship; and a line with a terminal bar (—|) represents inhibition. The gene labeled in red is HaWRKY33. The “?” symbol represents a pattern recognition receptor (PRR) that is yet to be identified or char-acterized.

Through KEGG analysis, we found that the gene *HaWRKY33* is involved in regulating the plant–pathogen interaction pathway (Figure 6). The MAPK cascade pathway is responsible for transmitting, amplifying, and conducting intracellular signals to the response genes of plant cells, stimulating the plant’s response to different signals, and playing a pivotal role in the plant’s defense response. In the plant–pathogen interaction pathway, extracellular pathogens are recognized by receptor proteins, and the information is transmitted into the cell via endocytosis, further activating downstream MPK4. These signals are then transmitted into the cell nucleus and conveyed to the transcription factor HaWRKY33, which further regulates the plant’s stress resistance.

#### 2.2.2. RT-qPCR Validation of Differentially Expressed Genes

**Figure 7:**
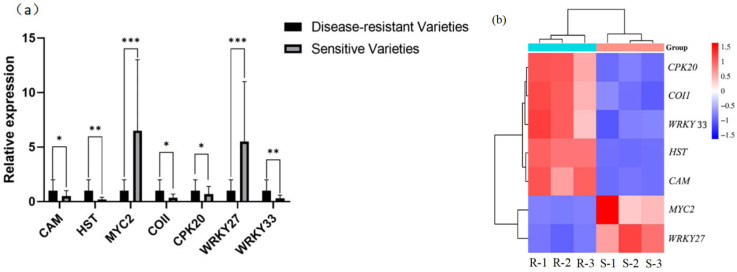
Seven differentially expressed genes were determined by RT-qPCR and transcriptome sequencing. (**a**) *** indicates *p*-value < 0.001; ** indicates *p*-value < 0.01; and * indicates *p*-value < 0.05. (**b**) R indicates disease-resistant varieties, and S indicates susceptible varieties, with three replicates per variety.

To verify the sequencing results of the transcriptome, the differentially expressed genes in sunflower mycosphaerella-resistant and susceptible varieties after inoculation with *S. sclerotiorum* were analyzed in detail, and the differentially expressed genes that were up-regulated and down-regulated in the resistant varieties relative to the susceptible varieties were selected as candidate genes, and seven genes were selected from the different gene function classifications to be verified by RT-qPCR. The results of RT-qPCR showed that the transcriptome sequencing results have high accuracy (Figure 7).

### 2.3. Bioinformatics Analysis of the HaWRKY33 Gene in Sunflowers

#### 2.3.1. Yeast Two-Hybrid Screening by HaWRKY33

Based on transcriptomic sequencing and RT-qPCR, it is speculated that *HaWRKY33* may regulate the defense response of sunflower against *S. sclerotiorum* through the plant-pathogen interaction pathway. Therefore, we performed a yeast two-hybrid screen using the gene *HaWRKY33* (*LOC110884429*), as shown in Table S3.

The *HaWRKY33* gene was inserted into the pGBKT7 vector to construct the pGBKT7-*HaWRKY33* fusion expression vector. “ORF” stands for open reading frame, which refers to a segment of nucleic acid sequence between the start codon and the stop codon that can encode a protein.

“ORF true” indicates that the cDNA fragment inserted into the library vector is in-frame with the reading frames of elements such as the transcription activation domain on the vector. This means that the amino acid sequence encoded by the cDNA can be expressed in the correct order with the transcription activation domain, forming a complete, potentially functional fusion protein.

“ORF not” indicates that the cDNA fragment inserted into the library vector is not in the correct reading frame with the elements on the vector, resulting in a misaligned (not in-frame) state. In this case, due to the altered reading frame, the amino acid sequence encoded by the cDNA may become disrupted, potentially leading to premature termination codons, resulting in incomplete expression or functional abnormalities of the fusion protein.

During subsequent bioinformatics prediction analysis, positive clones with a true ORF should be prioritized. This is because clones with true ORF ensure that the protein encoded by the cDNA is correctly fused with the transcription activation domain, and the expressed fusion protein is more likely to retain its natural structure and function, thereby more accurately reflecting the interactions between proteins.

#### 2.3.2. Prediction of Candidate Interacting Proteins with HaWRKY33 by AlphaFold 3

The six proteins, whose cDNAs were fusion-coded in the library vector, were subjected to protein interaction prediction using AlphaFold 3 3.0.1 (accessed on 15 March 2025 via https://alphafoldserver.com/). After screening, the combined complex protein conformation prediction (ipTM and pTM) revealed that HaWRKY33 had a higher absolute value of docking scores with AOA251SVV7 and that the probability of binding between HaWRKY33 and AOA251SVV7 was high, as shown in Table 2.

#### 2.3.3. Prediction of Key Amino Acid Sites for HaWRKY33-AOA251SVV7 by PyMOL

**Figure 8:**
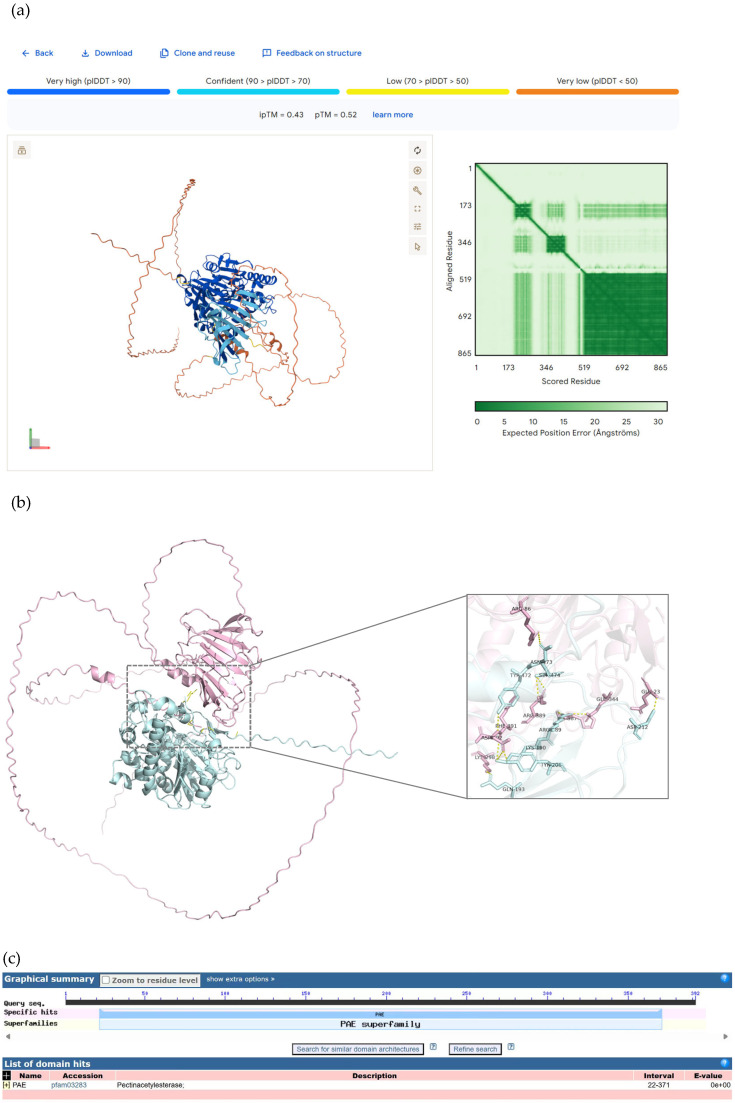
Protein conformation and conserved domains of HaWRKY33 and AOA251SVV7. (**a**) HaWRKY33-AOA251SVV7 interaction prediction analysis using AlphaFold 3. (**b**) Visualization of HaWRKY33-AOA251SVV7 molecular docking. (**c**) Conservative domains of HaWRKY33 and AOA251SVV7.

AlphaFold 3 was used to perform protein–protein interaction conformation prediction analysis on HaWRKY33-AOA251SVV7; the iPTM + PTM value was 0.96 > 0.75, as shown in Figure 8a. PyMOL was used to visualize the key amino acids at the protein interface of HaWRKY33-AOA251SVV7, as shown in Figure 8b. The NCBI (https://www.ncbi.nlm.nih.gov/) Molecular Biology Database was applied to analyze the conserved domains of HaWRKY33 and AOA251SVV7, as shown in Figure 8c. Among them, the amino acids GLN-193 and ARG-189 in HaWRKY33 are located within the conserved domain, and the corresponding amino acids LYS-298 and GLU-344 in the protein AOA251SVV7 are also within the conserved domain of that protein, as shown in Table 3. Therefore, we speculate that GLN-193 and ARG-189 in HaWRKY33 may be key amino acid sites for disease resistance.

In protein engineering, when inducing the greatest possible change in the three-dimensional structure of a protein through bioinformatic mutation simulation, it is necessary to select replacement amino acids that differ most significantly from the original amino acids in terms of physical and chemical properties. The selected amino acids for mutating GLN-193 and ARG-189 are shown in Table 4.

**Figure 9:**
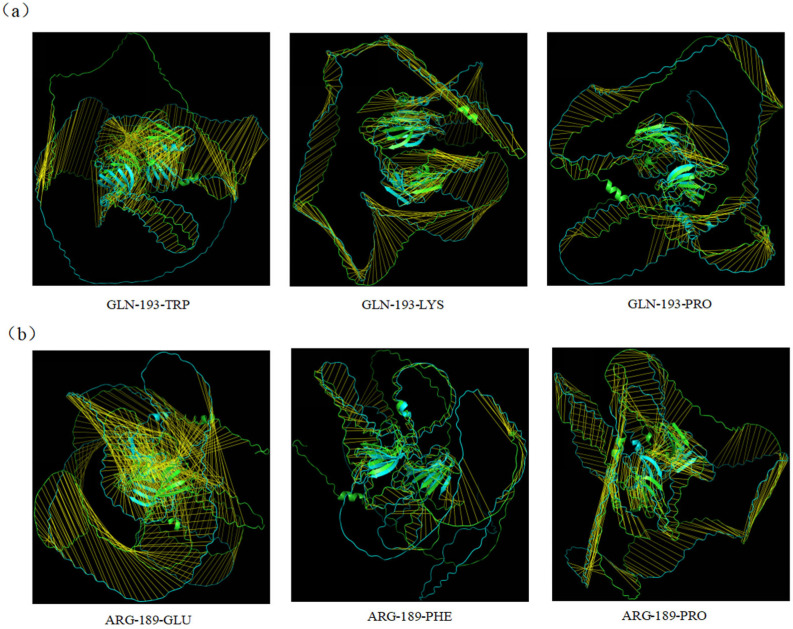
Three-dimensional structure comparison diagram of HaWRKY33 proteins after bioinformatic mutation simulation. (**a**) Comparison of the overall protein tertiary structures after the bioinformatic mutation of amino acid GLN-193 to TRP, LYS, and PRO. (**b**) Comparison of the overall protein tertiary structures after the bioinformatic mutation of amino acid ARG-189 to GLU, PHE, and PRO.

Through bioinformatic mutation simulation, we compared the three-dimensional structures of the HaWRKY33 protein before and after mutation using PyMOL, as shown in Figure 9. After site-directed mutagenesis of the protein, the root mean square deviation (RMSD) is a commonly used indicator to measure the degree of change in the three-dimensional structure before and after mutation. RMSD calculates the atomic position differences between the mutated protein and the wild-type protein; the larger the value, the more significant the structural change. We analyzed the RMSD values of HaWRKY33 after bioinformatic mutation simulation using PyMOL, as shown in Table S4. Among these, the RMSD value for the mutation of amino acid ARG-189 to GLU was the largest, indicating the most significant structural changes in the HaWRKY33 protein after mutation. Therefore, we speculate that amino acid ARG-189 may be a key amino acid site for disease resistance.

## 3. Discussion

WRKY transcription factors are a large class of transcription factors unique to plants [25], and although WRKY proteins share a lot of commonalities in structure and binding motifs and are related to each other, the physiological functions they are involved in are not regular, i.e., a WRKY transcription factor regulates different pathways simultaneously [26]. This may indirectly lead to the involvement of WRKY proteins in the regulation of several physiological processes in plants, such as the regulation of plant-specific secondary metabolic pathways [27,28], the response of plants to biotic (pathogen) and abiotic stresses (cold, heat, drought, etc.) [29], and the regulation of basic physiological processes (seed dormancy and germination, flowering, etc.) in plants [30]. Li experimentally verified that the expression of the *HaWRKY33* gene increased rapidly after a certain degree of inhibition at the early stage of sunflower rust infestation, indicating that the expression of this gene is strongly induced by rust infestation and that the rapid and timely response at the early stage of infestation in disease-resistant varieties may be an important factor involved in the resistance to the disease [21]. Zhang compared the resistance of plants overexpressing apple *MdWRKY33* to spotted leaf spot disease (*Alternaria alternata*) with that of silenced plants and found that the overexpression of *MdWRKY33* was less severe compared to the control, whereas the silenced plants were more severe compared to the control, which suggests that the expression of the *MdWRKY33* gene can significantly improve disease resistance [31]. WRKY transcription factors are involved in a variety of plant responses to adversity and play an important regulatory role in plant growth.

**Figure 10:**
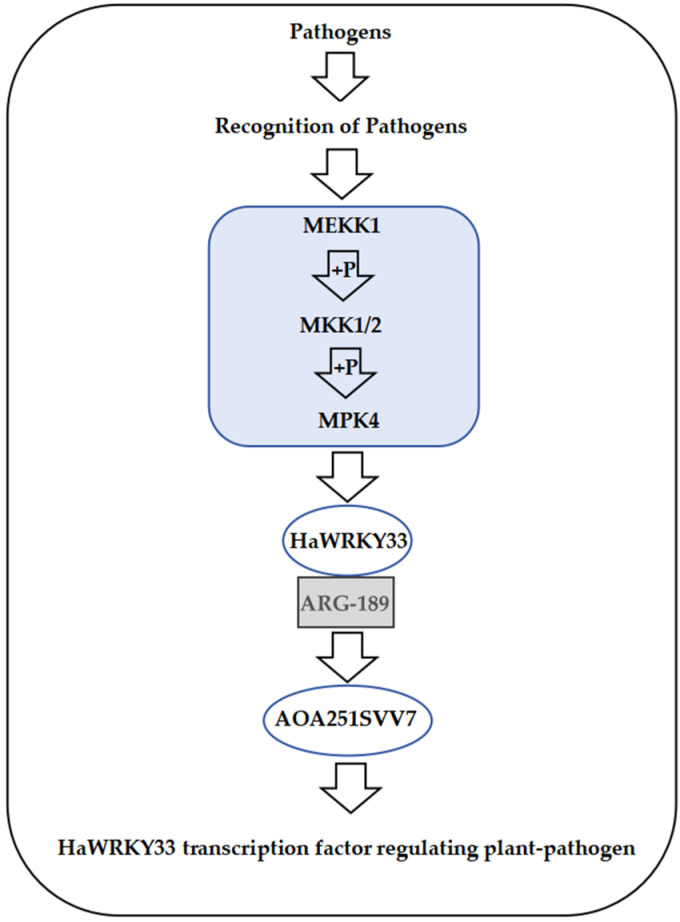
HaWRKY33-mediated regulatory network diagram.

In this study, we first collected a large amount of sunflower germplasm resources through field surveys and conducted phenotypic identification of disease resistance, finding that most varieties exhibited immune, moderately resistant, or resistant characteristics in the field. However, results from indoor inoculation experiments showed that most varieties became susceptible or moderately susceptible after inoculation. This discrepancy between field and indoor experiment results may stem from various factors, such as the complex field environment, where multiple biotic and abiotic factors interact, potentially triggering diverse disease resistance mechanisms in sunflowers. In contrast, indoor inoculation experiments have relatively simple conditions, simulating only the infection by a specific pathogen. In the current study, a notable progression has been observed and mentioned, specifically the shift from the identification of the dominant advantageous strain MCG1 to its subsequent application in indoor inoculation experiments. This transition represents a significant step in the research process, moving from a fundamental understanding of the microbial population structure to a more practical, experimental phase aimed at exploring the potential utility of this particular strain. Additionally, many interaction pathways between *S. sclerotiorum* and its hosts remain uncovered [32]. Research has confirmed that WRKY33 is involved in the regulation of various biological and abiotic stresses and occupies a central position in the regulatory network [33,34]. Against this backdrop of discrepancy, we identified the differentially expressed gene *HaWRKY33* through RNA-seq analysis combined with qRT-PCR technology, suggesting that this gene may play a crucial role in sunflower resistance to *S. sclerotiorum*. Interestingly, several wild *Helianthus* relatives have been identified, each displaying variable tolerance levels to sunflower disease. Nevertheless, none of the varieties display resistance, and differences in *Helianthus* ploidy levels hinder traditional resistance breeding [35]. Although field trials did not directly assess resistance to *S. sclerotiorum*, the differential expression of the *HaWRKY33* gene under different experimental conditions suggests that it may be involved in the regulatory network of sunflower resistance to multiple pathogens, demonstrating broad potential for disease resistance. Under stress signals such as pathogen infection, the proposed *HaWRKY33*-mediated regulatory model is shown in Figure 10.

Although this study achieved certain results, it still has some limitations. Although yeast two-hybrid and bioinformatics methods were used to predict the interacting proteins of HaWRKY33, these interactions require further validation in vivo experiments to determine their precise roles in the sunflower disease resistance signaling pathway. It is important to note that the functional validation of the key amino acid site ARG-189, as presented, is based solely on protein structure analysis and post-mutation conformational changes. At present, there is a lack of direct biological functional evidence to substantiate these findings.

CRISPR-Cas-mediated genome engineering presents unparalleled opportunities to engineer crop varieties cheaper, easier, and faster than ever [36]. Oil-producing plants are the world’s most significant crops, both economically and nutritionally. Despite this, numerous countries have looked into genetic engineering techniques for crop development. It initiated the success of genetically altered crop types for biotic (tolerance for herbicide and resistance to disease), abiotic (salinity, temperature, and heavy metals), and nutritious benefits [37]. It is therefore hypothesized that future studies could employ gene editing technology to create sunflower plants with targeted mutations at the ARG-189 site. Subsequently, phenotypic analysis and disease resistance assessment would be conducted to clarify the specific role of this site in the *HaWRKY33* disease resistance function. These predictive claims are proposed based on current knowledge and are subject to additional experimental validation to ensure their accuracy and reliability.

Beyond gene editing, complementary experimental techniques may be recommended to further elucidate protein–protein interactions. Co-immunoprecipitation (Co-IP) serves as a robust method for identifying interacting proteins within complex biological samples. This technique employs specific antibodies targeting proteins such as HaWRKY33 to precipitate the target protein and its interacting partners from cell lysates. Immunoprecipitated proteins can be analyzed by mass spectrometry to identify interacting molecules. This approach can validate whether AOA251SVV7 and *HaWRKY33* physically interact in vivo and potentially identify unknown interacting proteins involved in the same signaling pathway.

Another valuable technique is the Two-Fluorescent Complementation (BiFC) assay. In BiFC experiments, two target proteins (HaWRKY33 and AOA251SVV7) are each fused to a non-fluorescent fragment of a fluorescent protein, such as Yellow Fluorescent Protein (YFP). When these fusion proteins interact in vivo, the non-fluorescent fragments reassemble into functional fluorescent proteins detectable by fluorescence microscopy. This method not only confirms physical interactions between proteins but also provides subcellular localization information, which is crucial for understanding the functional context of interactions.

By integrating gene editing, co-immunoprecipitation, and bimolecular fluorescence complementation (BiFC) approaches, researchers have proposed a more comprehensive and reliable strategy to overcome existing research limitations. This multi-pronged method not only addresses the lack of direct biological evidence regarding ARG-189 protein interactions but also significantly advances our understanding of the molecular mechanisms underlying HaWRKY33-mediated disease resistance in sunflowers.

## 4. Materials and Methods

### 4.1. Identification of Sunflower Resources Resistant to S. sclerotiorum

#### 4.1.1. Sunflower Head Rot Field Identification Criteria

We collected 426 sunflower lines, of which 289 were from the Sunflower Technical Service Center of Gannan County, 82 were from the national oil system, 30 were from other research institutes, and 25 were from the national germplasm resource bank. The test site is the experimental field of the Gannan County Sunflower Technology Service Center. The soil used in the experiment was chernozem. Each sunflower line was planted in plots consisting of 5 rows, with a spacing of 50 cm between plants and a total of 65 rows. Within each plot, there were 50 plants. Disease surveys were conducted throughout the disease season to record the number of flower heads at each disease level. The disease index was calculated based on these observations, allowing for the evaluation of the resistance of different sunflower varieties (Table 5). The disease classification standard is as follows: grade 0: asymptomatic; grade 1: the lesion on the back of the head accounted for less than 25% of the head area; grade 2: the lesion area on the back of the head accounted for 26–50% of the head area; grade 3: the lesion area on the back of the head accounted for 51–75% of the head area; and grade 4: the lesion area on the back of the head accounted for more than 75% of the head area [38]. Data calculation is expressed as follows:

$$\mathrm{Disease}\;\mathrm{index}=\frac{\sum \mathrm{Number}\;\mathrm{of}\;\mathrm{sunflower}\;\mathrm{heads}\;\mathrm{at}\;\mathrm{all}\;\mathrm{levels}\times \mathrm{Relative}\;\mathrm{level}\;\mathrm{value}}{\mathrm{Total}\;\mathrm{number}\;\mathrm{of}\;\mathrm{heads}\;\mathrm{surveyed}}\times 100$$

#### 4.1.2. Indoor Identification for Sunflower Head Rot Disease

According to the results of field identification, the lines that grew well in the field of Gannan County, with strong stems and high yields, were selected and planted in the artificial climate chamber and cultured under the conditions of 50–60% relative humidity and 25 °C. The local dominant races obtained from genetic diversity analysis were inoculated onto sunflower heads. The inoculation was carried out by the mycelial plug attachment. At the early stage of flowering, we created wounds on sunflower heads, attached mycelial plugs, and bagged heads. After 4 weeks, the incidence of each head was investigated and recorded, and the disease index was calculated. The calculation method is the same as the field investigation.

### 4.2. Research on the Genetic Diversity of S. sclerotiorum and Methods for Predicting Dominant Strains

#### 4.2.1. Isolation and Purification of *S. sclerotiorum*

The infected sunflower flower heads (*S. sclerotiorum*) were collected (Table S5), and the pathogen was isolated using tissue separation methods [39]. Tissue samples were taken from the diseased edges of the sunflower flower heads. The specific procedure is as follows: The ultra-clean workbench was pre-sterilized under ultraviolet light for 15–20 min. Under sterile conditions, the tissue was disinfected with 70% ethanol for 1 min, then immersed in 5% sodium hypochlorite solution for 30 s, followed by three rinses with sterile water and dried with sterile filter paper. The treated tissue was placed in the center of PDA medium, sealed, and incubated at 25 °C in an incubator. Once the mycelium has completely covered the culture dish, use a punch to collect a mycelium block from the outer edge of the colony, transfer it to a new PDA medium, and repeat the isolation and culture process three times. Once the third isolated strain has completely covered the medium, store it in a 4 °C refrigerator for future use.

#### 4.2.2. Identification of the Affinity of *S. sclerotiorum* Mycelium

Disease resistance identification is the main way to carry out screening of germplasm resources for disease resistance and breeding for disease resistance, but since sunflowers can be infected with *S. sclerotiorum* at all growth stages and have a wide range of disease types, it is very difficult to guide the identification of resistance to *S. sclerotiorum* in sunflowers. The mycelial inoculation method is to isolate and purify the disease samples or nuclei collected in the field, then cultivate the mycelium on the culture medium, and then inoculate the mycelium of *S. sclerotiorum* to the roots, stems, or flower disks of sunflowers for resistance identification [40,41]. For the MCGs, mycelial plugs (5 mm in diameter) were obtained from the colony edge of each *S. sclerotiorum* isolate after 5–7 days of growth. Three different plugs were placed in one Petri dish with PDA medium as a pairing. Three mycelium plugs were arranged in an equilateral triangle in the middle of the Petri dish, and the distance between the edge of the Petri dish was 2 cm. Each combination was repeated three times. After inoculation, the isolates were cultured in the dark at 22 °C. After 7–10 days, the affinity of the isolates in the Petri dish was observed and recorded. Repeat three times for each group. According to the classification method of Kull et al. (2004) [41], the fusion of isolates was divided into affinity and incompatibility (the affinity condition was that there was no necrosis line, blank, or mycelial uplift area in the interaction area between isolates; the incompatibility condition was an interaction area between isolates with a necrosis line, blank, or mycelium uplift area).

#### 4.2.3. Assessment of SSR Genetic Diversity in *S. sclerotiorum* Strains from Sunflower

The simple sequence repeat markers (SSR) method was created by Moore in 1991. Eukaryotic organisms have many simple repeats of 2–5 bp, called “microsatellite DNA” [42,43,44]. Compared to other molecular markers, SSR molecular markers are characterized by lower cost, less DNA requirement, and good reproducibility [45,46]. We used the primers to detect microsatellite loci to characterize the genetic structure of 66 *S. sclerotiorum* of sunflower head rot (Table S2). These primers were developed by Sirjusingh and Kohn [47].

#### 4.2.4. Statistical Analysis of Genetic Diversity and Inference of Dominant Varieties

Based on the results of polyacrylamide gel electrophoresis, amplified polymorphic bands were statistically analyzed. The presence or absence of amplified bands was recorded as “1” and “0”, respectively, with stable and easily identifiable polymorphic bands designated as “1” and bands lacking polymorphism or gaps recorded as “0”. During statistical analysis, a few non-repeatable bands were disregarded. Cluster analyses of SSR marker data were performed using Jaccard’s similarity coefficient and the unweighted pair group method with arithmetic mean (UPGMA) in NTSYS software (version 2.02). The determined mycelium affinity groups were compared with the cluster analysis results. The branching patterns of strains within the largest affinity group in the clustering results were observed to infer the dominant race in the local population.

### 4.3. Transcriptome Sequencing Analysis

#### 4.3.1. Sample Collection

The dominant strain of *S. sclerotiorum* identified through separation and identification was activated on PDA medium. Once the mycelium had completely covered the culture dish, the mycelium was inoculated onto sunflower flower heads using the mycelium block attachment method. First, make a 1 cm long semicircular incision near the edge on the underside of the receptacle using a dissecting blade. Insert a 3 mm^2^ mycelium plug into the incision, or directly insert a mycelium plug into the edge of the receptacle’s underside for inoculation [48]. After inoculation, the flower heads were placed in bags and continued to be cultivated in an artificial climate chamber. Thirty days after inoculation, mixed samples were collected from the flower heads of both disease-resistant and susceptible varieties, with each mixed sample weighing 0.1 g, and three replicates were performed. The samples were rapidly frozen in liquid nitrogen and stored at −80 °C. The samples were sent to OE Biotech Co., Ltd. (Shanghai, China) for transcriptomic sequencing analysis, and the preliminary results obtained were subjected to further analysis.

#### 4.3.2. RNA Extraction and Library Construction

Total RNA was extracted from sunflowers using the Trizol reagent according to the manufacturer’s instructions. RNA purity and quantification were assessed using a NanoDrop 2000 spectrophotometer (Thermo Fisher Scientific, Wilmington, DE, USA), and RNA integrity was evaluated using an Agilent 2100 Bioanalyzer (Agilent Technologies, Santa Clara, CA, USA). Transcriptome libraries were constructed using the VAHTS Universal V5 RNA-seq Library Prep Kit (Vazyme Biotech, Nanjing, China) according to the instructions.

#### 4.3.3. RNA Sequencing and Differential Gene Expression Analysis

With the rapid development of next-generation high-throughput sequencing technology, transcriptome sequencing technology, i.e., RNA-seq, is also developing rapidly. Compared with the traditional technology, RNA-seq does not require the design of probes, and it can quantitatively detect the expression of RNA [49,50,51]. Sunflower samples resistant and susceptible to *S. sclerotiorum* infection were compared. Library sequencing was performed using the Illumina NovaSeq 6000 sequencing platform, generating 150 bp paired-end reads. Each sample yielded approximately 46–51 raw reads. Raw reads in FASTQ format were processed using fast software to remove low-quality reads, yielding clean reads for subsequent data analysis. Transcriptome sequencing and analysis were performed by OE Biotech Co., Ltd. (Shanghai, China). HISAT2 2.1.0 software was used for reference genome alignment, and gene expression levels (FPKM) were calculated. HTSeq-count was used to obtain the read counts for each gene. Finally, DESeq2 1.25.9 software was used to screen for differentially expressed genes (DEGs), defining genes with a q-value < 0.05 and a fold change > 2 or fold change < 0.5 as DEGs.

#### 4.3.4. Differentially Expressed Gene Analysis

To functionally characterize the differentially expressed genes, Gene Ontology (GO) enrichment analysis was carried out to identify significantly over-represented biological processes, molecular functions, and cellular components. Count the number of differentially expressed genes in each GO term and use the hypergeometric distribution algorithm to calculate the significance of gene enrichment in each GO term, yielding a *p*-value. A lower *p*-value indicates greater statistical significance. KEGG is the primary public database for pathways. We performed pathway analysis on differentially expressed protein-coding genes using the KEGG database (combined with KEGG annotation results) and calculated the significance of gene enrichment in each pathway entry using the hypergeometric distribution test.

#### 4.3.5. Quantitative Real-Time PCR Validation

For RT-qPCR analysis, we obtain the relevant gene sequences from the sunflower reference genome, design primers using NCBI Primer-BLAST, and amplify products with a length of 150 bp or less. The kit used was THUNDERBIRD^®^ Next SYBR^®^ qPCR Mix (Toyobo, Osaka, Japan). Following the instructions, RT-qPCR reactions were performed on cDNA from sunflower disease-resistant and disease-susceptible flower disk tissue samples 48 h after inoculation with the pathogen. The amplification program was as follows: 95 °C for 1 min, 95 °C for 5 s, and 60 °C for 30 s, all for 40 cycles. The generated gene expression data were analyzed and processed using the 2^−ΔΔCt^ method, which determines relative changes in gene expression compared to control conditions. Data were obtained using one-way analysis of variance with GraphPad Prism 8 software (accessed on 9 January 2025 via https://www.graphpad.com/). The RT-qPCR primer information is shown in Table S6.

### 4.4. Bioinformatics Analysis of Genes Using AlphaFold 3 and PyMOL Software

#### 4.4.1. AlphaFold 3 Prediction of Protein Interaction Site Analysis

We employed the AlphaFold 3 3.0.1 (accessed on 15 March 2025 via https://alphafoldserver.com/)deep learning framework in conjunction with multi-modal biomolecular modeling techniques to systematically establish a predictive framework for protein interaction sites. By integrating protein three-dimensional conformations with modeling algorithms, the method achieves high-precision structural predictions for protein–protein and protein–nucleic acid complexes. Experiments demonstrate that this method achieves a confidence threshold of ipTM + pTM > 0.75 in gene–protein complex prediction, with significantly improved accuracy in predicting hydrogen bond networks at key amino acid sites compared to traditional molecular docking methods.

#### 4.4.2. Molecular Docking and Bioinformatic Mutation Simulation Analysis Using PyMOL

Molecular docking simulation was combined with site-directed mutagenesis analysis through a standardized workflow. Utilizing the molecular visualization and scripting capabilities of PyMOL 2.5.7, combined with docking algorithms, the workflow enabled precise conformation prediction and key residue identification for key crop functional proteins (e.g., disease-resistant proteins and metabolic enzymes) and target proteins (pathogen effector proteins and substrate molecules), providing an efficient computational tool for crop molecular breeding. Bioinformatic mutation simulations are generated using the mutation module built into PyMOL software. This process involves replacing the side-chain atomic coordinates of specific amino acid residues and performing energy minimization optimization on the mutated local structure to eliminate local steric hindrance. This simulates the spatial conformation of the protein after actual mutation, bringing the mutated structure closer to its physiological state. It provides a theoretical model foundation for subsequent analysis of how mutations affect protein structure and function.

## 5. Conclusions

We conducted a field survey of sunflowers in Gannan County, Qiqihar City, collecting a total of 426 sunflower germplasm resources for field-based disease resistance phenotyping. Most varieties exhibited immune, moderately resistant, or resistant traits in the field. Subsequently, sunflower varieties with excellent comprehensive field traits were selected for indoor inoculation testing. The results indicated that most varieties became susceptible or moderately susceptible after inoculation. RNA-seq analysis was performed on mixed samples of three moderately resistant and three susceptible sunflower varieties after inoculation with *S. sclerotiorum*. Combining RNA-seq and RT-qPCR technologies, the differentially expressed gene *HaWRKY33* was identified. A yeast two-hybrid screening library was constructed, and protein interactions were predicted using AlphaFold 3. Among these, the protein AOA251SVV7 scored highly and showed good docking results. We visualized the protein interaction structure between HaWRKY33 and AOA251SVV7 using PyMOL, visualized the key amino acid residues at the protein interface, and performed bioinformatic mutation simulation on the amino acid ARG-189-GLU of sunflower HaWRKY33. The RMSD values were the highest before and after the mutation, and the changes in the three-dimensional conformation of the protein were also the greatest. Therefore, we speculate that ARG-189 may be the key amino acid site for HaWRKY33 disease resistance, providing a theoretical basis for further elucidating the disease resistance mechanism of sunflowers in the future.

In summary, we revealed the potential importance of the *HaWRKY33* gene in sunflower disease resistance through comprehensive experiments and analyses, identified and predicted its interacting proteins and key amino acid sites, and provided new insights and a theoretical basis for understanding sunflower disease resistance mechanisms. Future research will further expand and deepen these findings, providing more effective strategies and targets for sunflower disease resistance breeding.

## Figures and Tables

**Table 1:** Statistical information of different mycelial compatibility groups.

MCGs	Number of Isolates	Isolate Designations
1	21	1, 16, 18, 26, 27, 28, 29, 30, 31, 33, 35, 36, 37, 38, 40, 41, 39, 34, 49, 50, 62
2	8	42, 43, 44, 45, 46, 47, 48, 51
3	11	2, 3, 4, 5, 6, 7, 11, 13, 15, 19, 24
4	9	8, 9, 10, 12, 14, 17, 20, 21, 32
5	11	22, 23, 25, 52, 53, 54, 55, 60, 61, 63
6	7	56, 57, 58, 59, 64, 65, 66

MCGs = mycelial compatibility groups.

**Table 2:** Six proteins predicted by AlphaFold 3 for ipTM and pTM values.

Gene Name	Protein Name	Genebank	iPTM	PTM
DEAD-box ATP-dependent RNA helicase 17	A0A251V503	XP_022027146.1	0.19	0.4
pectin acetylesterase 8-like	A0A251SVV7	XP_022001922.1	0.43	0.52
uncharacterized protein LOC110937345 isoform X2	A0A251V192	XP_022035446.1	0.39	0.34
glucan endo-1,3-beta-glucosidase-like	A0A251TFA3	XP_021994768.1	0.26	0.46
putative elongation factor 1-alpha	EF1A	OTG20831.1	0.15	0.39
putative ribosomal protein L10P	AOA251TCN0	OTG08828.1	0.16	0.29

**Table 3:** Prediction of HaWRKY33-AOA251SVV7 interacting amino acids.

HaWRKY33	AOA251SVV7
ASN-473	ARG-86
SER-474	ARG-389
TYR-472	ASP-392
TYR-206	
LYS-190	
LYS-190	PHE-391
GLN-193	LYS-298
ASP-212	GLU-23
ARG-189	GLU-344
	HIS-387

**Table 4:** Candidate amino acid residues for bioinformatic mutation simulation at positions GLN-193 and ARG-189.

Amino Acid	Mutated Amino Acid	Reason
GLN-193	GLN-193-TRP	TRP is a hydrophobic amino acid that may disrupt existing hydrogen bond networks or hydrophilic interactions.
	GLN-193-LYS	LYS is positively charged (-NH_3_^+^) and may disrupt the original electrostatic equilibrium.
	GLN-193-PRO	The ring structure of PRO limits the φ angle, which may disrupt the α-helix or β-fold.
ARG-189	ARG-189-GLU	May damage salt bridges or cause electrostatic repulsion.
	ARG-189-PHE	Introducing hydrophobic mutations in key regions of positively charged residues disrupts protein–ligand binding or protein–protein interactions.
	ARG-189-PRO	The ring structure of PRO may disrupt α-helices or β-folds.

**Table 5:** Resistance evaluation criteria of sunflower lines.

**Resistance Evaluation**	**Disease Index**
I	0
R	<10
MR	10.1~30
MS	30.1~50
S	50.1~75
HS	>75.1

Types of disease resistance include I = immunization; R = resistance; MR = moderate resistance; MS = moderate susceptibility; S = susceptibility; and HS = high susceptibility.

## Data Availability

The data presented in this study are available from the corresponding author upon reasonable request.

## Supplementary Materials

The following supporting information can be downloaded at: https://www.mdpi.com/article/10.3390/plants14193018/s1. Table S1: The SSR markers for genetic diversity analysis; Table S2: The identification of 426 sunflower lines in experimental field and 148 sunflower lines indoor; Table S3: pGBKT7-HaWRKY33 screen library positive clone annotation; Table S4: RMSD values and HaWRKY33 point mutations for GLN-193 and ARG-189; Table S5: The 66 strains of *S. sclerotiorum* isolated from different sunflower lines in September 2022; Table S6: RT-qPCR primer information; Figure S1: Agarose electrophoresis by primer AF377919. 1-66 means the number of *S. sclerotiorum* isolates; Figure S2: Polyacrylamide gel electrophoresis by AF377921. 1-66 means the number of *S. sclerotiorum* isolates. Figure S3: Top 30 items of GO enrichment analysis of differentially expressed genes. The vertical axis in the figure represents the GO term names. In panels (a) and (b), the horizontal axis represents the number of differentially expressed genes. In panels (c) and (d), the horizontal axis represents the −log_10_ *p*-value. Figure S4: KEGG enrichment analysis of top 20 differentially expressed genes. (a) KEGG enrichment results of up-regulated differentially expressed genes in resistant cultivars compared to susceptible cultivars. (b) KEGG enrichment results of down-regulated differentially expressed genes in resistant cultivars compared to susceptible cultivars. The horizontal axis in the figure represents the number of genes. CellP: Cellular Processes; EnvIP: Environmental Information Processing; GenIP: Genetic Information Processing; Metab: Metabolism; OrgaS: Organismal Systems.
