# Supplementary material for: Putative Transcriptional Regulation of HaWRKY33-AOA251SVV7 Complex-Mediated Sunflower Head Rot by Transcriptomics and Proteomics
Source: Plants (Basel). 2025 Sep 29;14(19):3018. doi: 10.3390/plants14193018 (PMC12526259; doi:10.3390/plants14193018)
Supplement: Supplementary file 1 [file plants-14-03018-s001.zip › plants-3858401-supplementary.pdf]

Table S1. The SSR markers for genetic diversity analysis

GenBank accession	Repeat motif	Primer sequences (5′-3′)	Ta[y] (°C)	Amplicon size (bp)	No. of alleles	Polymorphic fragments[Z]
AF377899	(TA)5 and (CA)10	CCGAGCATAATATACATCC AAGGTTATATTTCCCTCGC	50	490-504	4	N
AF377900	(GT)8	GTAACACCGAAATGACGGC GATCACATGTTTATCCCTGGC	55	318-325	3	3
AF377901	(TTTTTC)2(TTTTTG)2(TTTTTC)	GGGGGAAAGGGATAAAGAAAAG CAGACAGGATTATAAGCTTGGTCAC	55	479-484	2	2
AF377902	(GA)14	TTTGCGTATTATGGTGGGC ATGGCGCAACTCTCAATAGG	55	160-172	4	3
AF377903	(CA)9(CT)9	GCCGATATGGACAATGTACACC TCTTCGCAGCTCGACAAGG	55	358-382	4	N
AF377905	(GA)6GG(GA)6(GGGA)2	CTTTCCTTTCGTTTGAGGG GGCAGGTAATGTTGCTTGG	55	276-284	3	N
AF377906	(CA)9	CGATAATTTCCCCTCACTTGC GGAAGTCCTGATATCGTTGAGG	55	215-225	4	N
AF377907	(GTGGT)6	TCTACCCAAGCTTCAGTATTCC GAACTGGTTAATTGTCTCGG	55	284-304	4	4
AF377908	[(GT)2GAT]3(GT)14GAT(GT)5[GAT(GT)4]3(GAT)3	CAGACGAATGAGAAGCGAAC TTCAAAACAACGCTCCTGG	55	245-320	5	4
AF377909	GT10	CCTGATATCGTTGAGGTCG ATTTCCCCTCACTTGCTCC	55	202-212	5	4
AF377910	CA12	CACTCGCTTCTCCATCTCC GCTTGATTAGTTGGTTGGCA	60	251-271	4	3
AF377911	(TTA)9	TCATAGTGAGTGCATGATGCC CAGGGATGACTTTGGAATGG	55	345-390	5	5

AF377912	(GT)7GG(GT)5	GACGCCTTGAAGTTCTCTTCC CGAACAAGTATCCTCGTACCG	55	268-278	4	4
AF377913	(TG)10	CTTCTAGAGGACTTGGTTTTGG CGGAGGTCATTGGGAGTACG	60	384-388	3	3
AF377914	CA6(CGCA)2CAT2	GAATCTCTGTCCCACCATCG AGCCCATGTTTGGTTGTACG	60	415-429	2	2
AF377916	GA9	GGTCTCATACAGTCTACACACA CTCTAGAGGATCTGCTGACA	60	410-414	3	3
AF377917	CA7(TACA)2	CCCTACAATATCCCATGGAGTC CCTCGTCTATCCGTCCATC	60	419-527	2	N
AF377918	TACA10	GTTTTCGGTTGTGTGCTGG GCTCGTTCAAGCTCAGCAAG	60	173-221	7	7
AF377919	(CT)12	TCGCCTCAGAAGAATGTGC AGCGGGTTACAAGGAGATGG	60	374-378	3	3
AF377921	(CATA)25	TGCATCTCGATGCTTGAATC CCTGCAGGGAGAAACATCAC	55	491-571	10	8
AF377922	(TATG)9	ATCCCTAACATCCCTAACGC GGAGAATTGAAGAATTGAATGC	55	362-378	5	4
AF377923	(AGAT)14(AAGC)4	GCTCCTGTATACCATGTCTTG GGACTTTCGGACATGATGAT	55	351-391	8	5
AF377924	(TAC)6C(TAC)3	TCAAGTACAGCATTTGC TTCCAGTCATTACCTACTAC	48	376-388	2	2
AF377925	(GTAT)6 and (TACA)5	GTAACAAGAGACCAAAATTCGG TGAACGAGCTGTCATTCCC	60	369-391	3	3
AF377926	(GTAA)2(GCAA)(GTAA)3	CTCATTTCATCCCATCTCTCC AATTCAAGCCTTCCTCAGCC	55	402-422	2	2

[Z]N = indicate the primer sets were not used for Simple Sequence Repeats of all 66 isolates ;

[y]Ta = annealing temperature.

Table S2. The identification of 426 sunflower lines in experimental field and 148 sunflower lines indoor.

Sunflower lines	Field identification[z]	Indoor identification[y]	Sunflower lines	Field identification	Indoor identification
N344	MR	N	NOL389	I	HS
FK6	R	N	NOL390	I	HS
S49	I	N	NOL395	I	HS
N354	I	N	NOL97	I	N
S11	R	N	NOL399	MR	N
N393	MR	N	NOL3102	I	HS
N57	R	N	NOL3103	I	N
N5	R	N	NOL3104	R	N
SO12	I	N	NOL3105	I	N
S255	I	N	NOL3106	I	N
S9	I	N	NOL3108	I	N
S141	MR	N	NOL110	I	HS
S22	I	N	NOL111	I	N
S339	I	N	NOL112	I	HS
S359	I	N	NOL338	I	N
N331	I	N	SW10	I	N
S25	MS	N	SW31	I	HS
N338	I	N	SW32	I	HS
N14	I	N	SW33	I	N
S359	I	N	SW34	I	N
S318	R	N	SW35	I	N
S397	I	N	SW36	I	N
S313	I	N	SW37	I	N
S311	I	N	SW38	I	N
S332	I	N	SW39	I	N
S29	R	N	SW310	R	N
S38	I	N	SW311	I	N
N32	R	N	SW312	I	HS
N342	I	N	SW315	I	N
N233	I	N	SW316	R	HS
S310	I	N	SW318	I	HS
N3113	I	N	SW319	I	HS
G338	I	N	SW323	I	HS
S321	I	N	SW324	I	N
S33	I	N	SW326	R	N
S35	I	N	SW328	S	HS
N110	I	N	SW327	I	N
N117	I	N	SW3291	I	HS
N112	I	HS	SW3292	I	HS
N122	I	HS	SW3293	I	HS
N125	MR	HS	SW331	R	HS
N129	I	HS	SW332	R	HS
N137	I	HS	SW334	I	HS
N142	I	N	SW335	I	N
N146	I	HS	SW336	R	N
N151	I	HS	SW337	I	N
NS110	I	N	SW338	R	HS

NS115	MS	N	SW339	I	N
NS113	I	N	SW340	I	N
N21	I	N	SW341	R	HS
N22	I	HS	SW342	I	N
N23	MR	HS	SW343	I	N
N25	I	HS	SW344	I	N
N26	I	HS	SW345	I	HS
N27	I	HS	SW346	I	N
N28	MR	HS	SW348	I	HS
N210	I	N	SW350	I	N
N212	R	N	SW352	I	N
N213	I	N	SW353	I	N
N214	I	N	SW354	I	N
N216	I	HS	SW356	I	N
N2162	I	N	SW357	I	N
N220	I	HS	SW3112	R	N
N221	I	HS	SW358	R	HS
N222	I	HS	SW359	I	HS
N223	I	HS	SW361	I	HS
N225	I	HS	SW362	I	N
N227	I	N	SW363	I	N
N228	I	N	SW365	I	N
N255	I	HS	SW368	R	N
N229	I	HS	SW369	I	N
N230	R	N	SW371	I	N
NT217	I	N	SW372	I	N
N233	I	N	SW374	I	HS
N236	I	HS	SW375	I	N
N237	I	N	SW376	I	N
N238	I	HS	SW377	I	N
N239	R	HS	SW378	I	N
N241	I	HS	SW380	R	HS
N242	MR	N	SW381	R	N
N245	MR	N	SW384	I	N
N246	I	N	SW386	I	HS
N2463	R	HS	SW387	R	HS
N248	R	N	SW390	S	HS
N249	I	HS	SW391	I	N
N251	MR	MR	SW392	R	HS
N2513	R	N	SW393	I	N
S223	R	N	SW394	MR	HS
N2110	R	N	SW3941	R	N
S28	MR	N	SW395	I	HS
N217	MR	HS	SW396	R	HS
W07	I	HS	SW397	I	HS
W09	R	HS	SW3100	I	N
W24	I	HS	SW3101	MR	HS
W218	I	HS	SW3102	MR	N
W226	S	HS	SW3104	I	HS
W227	S	MR	SW3105	I	N
W298	MR	HS	SW3106	I	N

W344	MR	HS	SW3110	I	N
W105	MR	HS	SW3113	I	HS
W117	R	HS	SW3114	I	HS
Z03	I	HS	SW3115	MR	HS
Z11	I	HS	SW3116	I	HS
Z13	I	HS	SW3117	S	HS
Z17	MR	HS	SW3119	I	N
Z142	S	HS	SW3126	I	N
Z143	S	HS	SW3130	I	N
Z155	R	HS	SW3180	MR	HS
Z156	R	HS	SW330	R	N
Z273	I	HS	SW317	I	N
Z278	I	HS	SW370	R	HS
Z616	I	HS	G34	MR	N
Z645	R	HS	P19S20	I	N
Z63	I	HS	P19S25	I	N
Z64	I	HS	P19S13	R	N
Z238	I	HS	P19L15	MR	N
FK1770	I	N	P19L156	MR	N
FK0918	R	N	P19L1510	I	N
NKY-KY1	MR	HS	P19L1511	I	N
K111-61	I	HS	P18S66	I	N
K112-19	MR	N	PGN101	R	HS
K69-19	I	N	P22	I	N
376	I	HS	GSK170	I	N
LKZ2	MR	N	2022A	I	N
LKZ6	R	N	2022E	R	N
F51	I	N	2022F	S	HS
LKZ12	R	N	2022H	MR	HS
ZY1	R	N	2022J	MR	HS
ZY2	MR	N	2022L	I	N
C2201	S	N	2022W	R	MR
C2202	I	N	22NEP	I	HS
C2203	S	N	SR17	I	HS
JK202	I	N	G2201	MR	HS
JK118	I	N	G2202	I	N
JK203	I	N	G2204	I	HS
GL601	I	N	G2205	I	HS
GL0361	MR	N	G2206	R	N
MD1819	MR	N	G2208	I	N
BC2202-01	I	HS	G2209	I	HS
BC2202-02	I	HS	G2210	I	N
BC2202-03	I	MR	G2211	R	HS
BC2202-04	MR	HS	G2212	MR	HS
BC2202-05	R	HS	G2214	I	N
BC2202-06	I	HS	G2215	R	N
BC2202-07	I	MR	G2216	MR	HS
BC2202-08	R	HS	G2218	I	N
NOL1	I	N	G22119	MS	N
NOL2	I	HS	G2220	I	N
NOL31	I	N	G2221	R	N

NOL32	R	N	G2224	I	N
NOL5	I	N	G2225	I	N
NOL5O	I	HS	G2226	I	N
NOL71	I	HS	G2228	I	N
NOL72	I	N	G2229	I	HS
NOL73	I	N	G2231	R	N
NOL8	I	N	G2232	R	HS
NOL9	I	HS	G2233	R	HS
NOL10	I	N	G2235	I	HS
NOL12	I	N	G2236	I	N
FS14	I	N	G2238	I	HS
NOL15	I	N	G2239	I	N
NOL16	MR	N	G2240	R	HS
NOL17	I	N	G2245	R	HS
NOL18	I	N	G2246	MR	N
NOL20	I	N	G2247	I	N
NOL22	I	N	G2251	I	N
NOL23	I	N	G2254	MR	N
NOL24	I	N	G2255	R	N
NOL25	I	N	G2256	I	HS
NOL26	I	HS	G2257	R	N
NOL28	I	N	G2259	MR	HS
NOL29	I	HS	G2262	I	HS
NOL30	I	N	G2273	I	N
NOL31	I	N	P6754	I	N
NOL32	R	N	P1864	R	N
NOL33	I	N	P1841	I	N
NOL34	I	N	P19D135	MR	N
NOL35	I	N	P19D133	S	N
NOL37	I	N	P337	S	N
NOL372	I	HS	P1721	MR	N
NOL39	R	N	P1729	I	N
NOL40	I	N	P034	R	N
NOL41	MR	HS	P1744	MR	N
NOL342	I	HS	P1734	MR	N
NOL42	R	HS	P1730	I	N
NOL44	I	N	SR17207	I	N
NOL45	I	N	P22O33	MR	N
NOL47	I	N	P3771	I	N
NOL48	I	N	P17L58	MR	N
NOL351	I	N	P1396	S	N
NOL353	R	N	P11317	MR	N
NOL354	I	N	P341	S	N
NOL355	I	N	P353	MR	N
NOL57	I	N	P1777	MR	N
NOL61	I	N	P1756	I	N
NOL62	I	HS	P7745	R	N
NOL63	I	N	LJ188	R	N
NOL365	I	N	P5696	MS	N
NOL367	I	N	P1040	R	N
NOL368	I	N	P13253	MR	N

NOL69	R	N	P120	MS	N
NOL71	I	N	P1729	R	N
NOL73	R	HS	SR1772	I	N
NOL74	I	HS	P0399	R	N
NOL377	I	N	LJ368	MR	N
NOL378	I	N	P071	R	N
NOL379	I	N	P073	MR	N
NFS81	I	N	P072	MR	N
NOL381	I	N	P1732	R	N
NOL382	I	N	P1738	R	N
NOL384	I	N	P1741	R	N
NOL386	I	N	PF1	MS	N

[Z]Types of disease resistance, including I = immunization; R = resistance; MR = moderate resistance; MS = moderate susceptible; S = Susceptible; HS = high susceptible.

[y]N = no indoor inoculation identification was conducted.

Table S3. pGBKT7-HaWRKY33 screen library positive clone annotation

Gene Name	Genebank ID	ORF（true or not）
branchpoint-bridging protein-like	XP_022006597.1	not
hypothetical protein E3N88_37993	KAD2804616.1	not
aquaporin NIP2-1-like	XP_022011398.1	not
uncharacterized protein LOC110927304	XP_022026658.1	not
DEAD-box ATP-dependent RNA helicase 17	XP_022027146.1	true
catalase-like isoform X2	XP_021976738.1	not
fructose-bisphosphate aldolase 1, chloroplastic	XP_022002053.1	not
fructose-bisphosphate aldolase, cytoplasmic isozyme 1	XP_022012695.1	not
pectin acetylesterase 8-like	XP_022001922.1	true
protein ABHD17B-like	XP_022020796.1	not
uncharacterized protein LOC110937345 isoform X2	XP_022035446.1	true
glucan endo-1,3-beta-glucosidase-like	XP_021994768.1	true
zinc transporter 5-like	XP_022035927.1	not
gamma-glutamyltranspeptidase 3-like	XP_022027211.1	not
putative elongation factor 1-alpha	OTG20831.1	true
probable pectinesterase/pectinesterase inhibitor 61	XP_021974218.1	not
aquaporin PIP1-3-like	XP_022022901.1	not
putative ribosomal protein L10P	OTG08828.1	true
hypothetical protein HanPSC8_Chr16g0718311	KAJ0821289.1	not

Table S4. RMSD values and HaWRKY33 point mutations for GLN-193 and ARG-189

Amino Acid	Mutated Amino Acid	RMSD
GLN-193	GLN-193-TRP	31.854
	GLN-193-LYS	27.545
	GLN-193-PRO	16.996
ARG-189	ARG-189-GLU	39.506
	ARG-189-PHE	10.577
	ARG-189-PRO	27.392

Table S5. The 66 strains of *S. sclerotiorum* isolated from different sunflower lines in September 2022.

Isolate designations[z]	Sunflower lines	Isolate designations	Sunflower lines
1	W227	34	41-0
2	SW394	35	G2206
3	NOL32	36	P337
4	N248	37	Z142
5	NOL399	38	SW380
6	N22	39	G2245
7	Z155	40	G2216
8	P19L15	41	W344
9	SW341	42	N393
10	SW330	43	SW3115
11	N23	44	SW358
12	SW331	45	BC2202-04
13	SW359	46	N2463
14	S25	47	2022H
15	P341	48	SW328
16	2022F	49	SW332
17	W226	50	G2246
18	G2201	51	NOL3104
19	NS115	52	SW390
20	SW381	53	2022J
21	SW392	54	S11
22	N125	55	W09
23	2022W	56	NOL32
24	O1170	57	P1721
25	SW316	58	Z156
26	SW338	59	G2212

27	Z17	60	SW3101
28	W226	61	G2259
29	N25	62	SW387
30	W227	63	G2254
31	SW3102	64	NOL42
32	P19L156	65	G2229
33	P073	66	N217

Isolate designations[z] means that the values in the column are the numbers of 66 isolates of *S.sclerotiorum*, and the numbers in MCGs and SSRs are the same as those in this table.

Table S6. RT-qPCR primer information

Gene	Accession No.	Forward-primer (5'-3')	Reverse-primer (5'-3')
CAM	LOC110908317	TTTGATAAGGATGGAGATGGCTACA	GGCCTCTGTGGGGTTTTGTC
WRKY33	LOC110884429	AATAGCGTATGTTGGGAGTA	TAGGTGGTTGGGTGGTGG
HST	LOC110873433	TCCAACGGTTGATTACTCGCT	CATCTGAAGTGAGTTACCTGGGA
MYC2	LOC110878445	GCGAAAAACCTTGATGTTTCTGA	TGCTTTGACAAACGGACGAA
COI1	LOC110878047	GCTTCGCGGCTCAAAAAGTT	TCCCCAATTACGTTTCTGGTATCAA
CPK20	LOC110868452	GCAGGAAGAGGCTCACACTA	ACAGCTTGGGTTCAATCACTGT
WRKY27	LOC110885886	AGGCTCCCCTTATCCGAGAAATTA	CCACTTGCTTCCTTGCCGAG

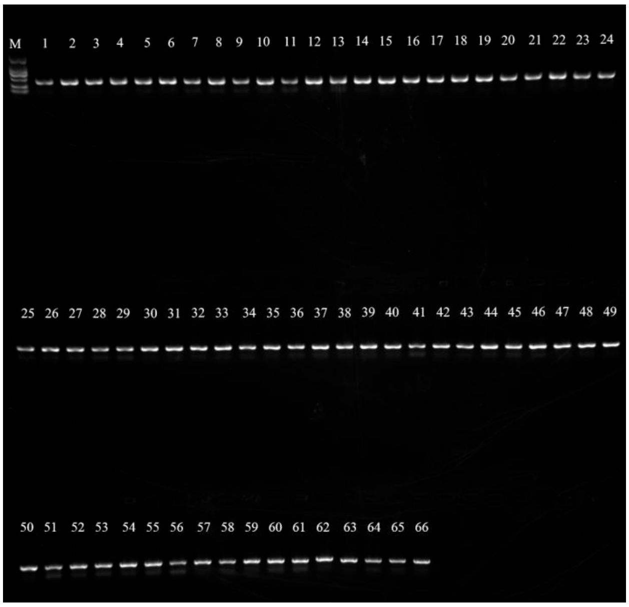

Figure S1. Agarose electrophoresis by primer AF377919. 1-66 means the number of *S. sclerotiorum* isolates.

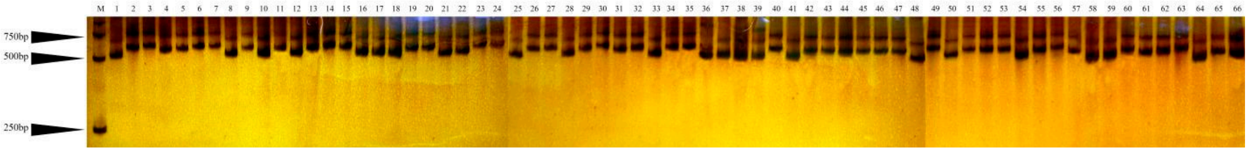

Figure S2. Polyacrylamide gel electrophoresis by AF377921. 1-66 means the number of *S. sclerotiorum* isolates.

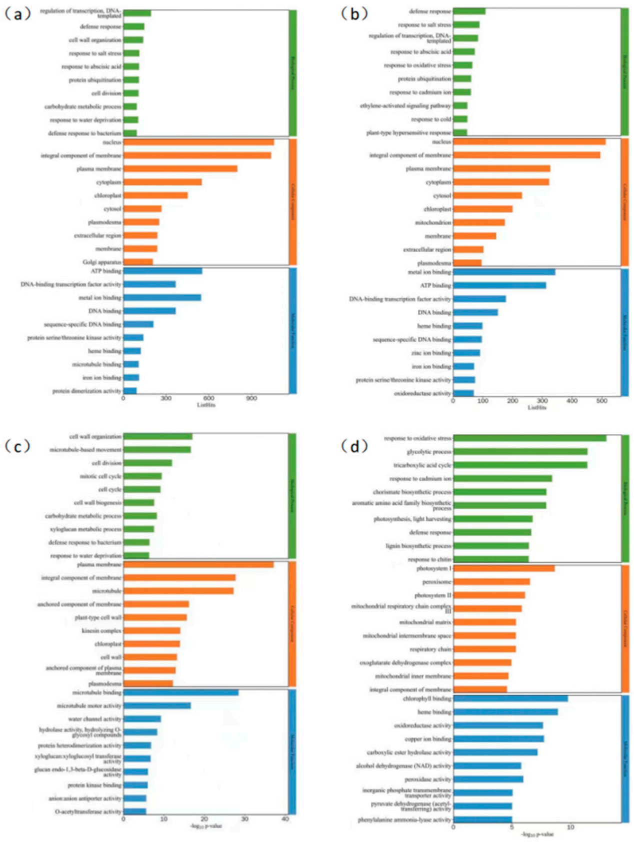

Figure S3. Top 30 items of GO enrichment analysis of differentially expressed genes. The vertical axis in the figure represents the GO term names. In panels (a) and (b), the horizontal axis represents the number of differentially expressed genes. In panels (c) and (d), the horizontal axis represents the $-\log_{10}$ p-value.

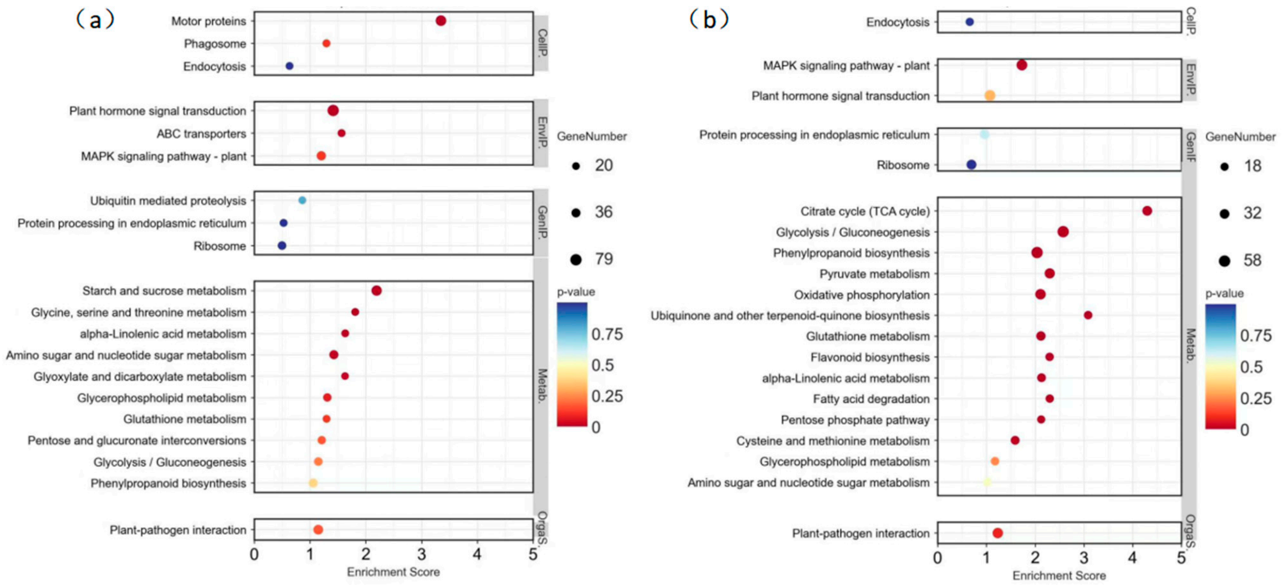

Figure S4. KEGG enrichment analysis of top 20 differentially expressed genes. (a) KEGG enrichment results of up-regulated differentially expressed genes in resistant cultivars compared to susceptible cultivars. (b) KEGG enrichment results of down-regulated differentially expressed genes in resistant cultivars compared to susceptible cultivars.The horizontal axis in the figure represents the number of

genes. CellP: Cellular Processes; EnvIP: Environmental Information Processing; GenIP: Genetic Information Processing; Metab: Metabolism; OrgaS: Organismal Systems.
